# Lactoferrin alleviates the adverse effects of early-life inflammation on depression in adults by regulating the activation of microglia

**DOI:** 10.1186/s10020-025-01094-9

**Published:** 2025-02-07

**Authors:** Wenli Wang, Qin An, Yunxia Zou, Yunping Dai, Qingyong Meng, Yali Zhang

**Affiliations:** 1 College of Food Science and Nutritional Engineering, China Agricultural University, Beijing, China; 2 The International Peace Maternity and Child Health Hospital, Shanghai Jiao Tong University School of Medicine, Shanghai, China; 3 College of Biological Sciences, China Agricultural University, Beijing, China

**Keywords:** Lactation, Lactoferrin, Inflammation, Depression, Innate immunity, Microglia

## Abstract

Lactation is a crucial phase of brain development, and the events and nutrients during this period have long-term consequences for the occurrence of depression. This study investigated the effect and mechanism of lactoferrin (LF) deficiency during lactation on depression in adulthood. Lactation LF-deficient mice were established by nursing wild-type mice using LF systemic knockout mother mice. Additionally, 14-day-old mice were injected with lipopolysaccharide (LPS) and subjected to chronic unpredictable mild stress when they reached 6 weeks of age. The results show that lactation lactoferrin deficiency increases depression-like behavior in adult mice, and the mechanism is associated with heightened neuronal damage, abnormal microglial activation, and decreased BDNF in the hippocampus. In contrast, recombinant human lactoferrin promotes neuronal proliferation by upregulating ERK 1 and 2 phosphorylation and attenuates LPS-induced neuronal injury and microglial activation by inhibiting the activation of Toll-like receptor 4—nuclear factor-kappa B pathway in vitro. Our findings suggest that lactoferrin intake during lactation protects neurons by regulating microglial activation, thereby effectively reducing depressive symptoms in adults.

**Supplementary Information:**

The online version contains supplementary material available at 10.1186/s10020-025-01094-9.

## Introduction

Neonates encounter a range of immune challenges, such as colitis, meningitis, sepsis, and pneumonia, owing to their immature immune systems (Vaagland et al. 2004). Inflammation during early life can have long-lasting effects on the development of the central nervous system (CNS) and influence subsequent behavior (Cao et al. 2021). Research has revealed that inflammatory events during brain development can greatly increase depression-like behaviors in adulthood (Cruz-Pereira et al. 2020). The period of lactation is vital for the growth of the immune and nervous systems, and the nutrients consumed during this stage have a significant impact on the infants' capacity to resist early inflammation, thereby having lasting effects on depression in adulthood (Owczarek et al. 2022; Marinho et al. 2018). Lactoferrin (LF), a significantly active whey protein found in breast milk, is recognized for its multifaceted health advantages, such as its capacity to regulate immune function, anti-inflammatory actions, and neurodevelopment (Bing 2016). Our previous investigation also demonstrated that lactoferrin deficiency during lactation results in decreased expression of genes associated with innate immunity and neurodevelopment in the hippocampus (Wang et al. 2023). However, there is a lack of research investigating the potential relationship between LF consumption during breastfeeding and the subsequent development of depression in later life. Furthermore, it is currently unclear whether lactating LF deficiency increases vulnerability to adult depression caused by early-life inflammation.

Microglia, which are the resident macrophages of the CNS, play a significant role in the pathogenesis of injury to the immature brains (Pierre et al. 2017). Numerous studies have provided substantial evidence for the activation of hippocampal microglia in both inflammatory and chronic unpredictable mild stress (CUMS)-induced depression (Pierre et al. 2017). Microglial activation is associated with depression through the suppression of neurogenesis and neuroplasticity. Microglial-released pro-inflammatory factors have been observed to exert detrimental effects on neurons and neurogenesis, whereas overactive microglia exhibit excessive phagocytosis and pruning of dendritic spines and synapses, resulting in aberrant neuronal function (Cao et al. 2021; Yirmiya et al. 2015). Furthermore, microglia have been implicated in the etiology of adult depression stemming from early inflammation. The initial inflammatory response triggers microglial activation and memory formation, which subsequently increases microglial sensitivity to inflammatory stimuli. Later in life, upon exposure to secondary stressors, microglia become hyperactivated and affect neuronal plasticity, neurogenesis, and other associated processes through abnormal pruning of synapses (Pierre et al. 2017). Research has demonstrated that inflammation during the early developmental stages triggers microglial vulnerability to subsequent stressful events via the chemokine (C-X3-C motif) receptor 1 protein. Furthermore, frequent microglial activation promotes excessive phagocytosis of dendritic spines, leading to a decrease in neuronal activity. Consequently, this decline in neuronal activation impairs the ability to cope effectively with stress-related challenges (Vaagland et al. 2004). However, only a few studies have examined the effects of exogenous LF on microglia. In a study conducted by Ginet et al. (2016) it was observed that microglia in the striatum exhibited diminished activation when subjected to LF treatment during lactation. Jun et al. have found that LF has the potential to mitigate neuropathic pain by inhibiting spinal microglial activation (Jun et al. 2016). However, there is currently a lack of research on the effect of LF on microglial activation in relation to depression.

In our study, we examined the consequences of early-inflammatory stimulation in lactating lactoferrin-deficient 14-day-old mice as well as the subsequent effects on depression following CUMS stimulation in adulthood. We also explored the protective properties of recombinant human lactoferrin (RHLF) against lipopolysaccharide (LPS)-induced neuronal damage and microglial activation in vitro. The results of our study suggest that the consumption of LF during lactation can mitigate the effects of early inflammation on adult depression by reducing neuronal damage, suppressing neuroinflammation, and regulating microglial activation. Our study demonstrates an innovative approach by illustrating that LF in early life reduces the risk of depression in adulthood through its neuroprotective and innate immune regulatory effects. This study underscores the importance of lactoferrin intake in infants during lactation and offers valuable insights into the early prevention and management of depression.

## Materials and methods

### Animals

The heterozygous LF gene knockout (KO) mice used in this study were obtained from Biocytogen Co., Ltd. (Beijing, China). Breeding to generate increased number of age-matched LF gene KO (*ltf*^*−/−*^*)* and wild-type (WT, *ltf*^+*/*+^) C57BL/6N mice. Prior to the start of the experiment, both *ltf*^*−/−*^ and *ltf*^+*/*+^ breeding pairs were synchronized to produce offspring at the same time. The litters born to *ltf*^*−/−*^ dams were replaced with age-matched pups born to *ltf*^+*/*+^ dams to form the KO-WT group on the second day postpartum. Similarly, WT-WT group was formed by standardizing control litters suckling *ltf*^+*/*+^ dams on the second day postpartum. During the suckling period, mice in the KO-WT group were fed with milk free of LF. The subsequent experiments involved the utilization of these two groups of mice. A total of 88 mice were used in this experiment. Every mouse was housed in a regulated environment followed with a 12:12 h light–dark pattern, a temperature of 22 ± 2 °C, humidity maintained at 60%, and 15–20 fresh air changes per hour. They were provided with free access to food and water. The guidelines of the Institutional Animal Care and Use Committee (SYXK 2020-0052) were followed for the treatment of all mice. Additionally, experiments were approved by the Animal Experimentation Committee of China Agricultural University (Beijing, China), the issue number is AW40702202-4-6.

### Early immune activation

Animals (wt-wt, ko-wt) were injected intraperitoneally at the age of 14 days with LPS [Sigma, L3755, Escherichia coli, serotype O26:B6, 50 μg/kg (Cao et al. 2021)] using an 1 mL insulin syringe (KDL, Shanghai). The control group animals did not any treatment. Offspring were weaned on day 21, males and females were placed in separated cages with four mice each on 4 weeks, male mice were selected throughout the next study.

### Procedure of CUMS

There are four groups of 6-week-old male mice included in the study: two groups without early immune activation (MWT-WT and MKO-WT) and two groups with early immune activation (Mwt-wt and Mko-wt). The CUMS detail and schedule were given in Table 1.

**Table 1:** Chronic unpredictable mild stress schedule

Stressors	Details	Days
Tail clamping	Tail pinch 1 cm apart from the end of the tail for 6 min	1, 10, 19, 23
Force swimming	Mice were placed for 6 min in a cylindrical clear plastic tank (30 cm high * 10 cm diameter) filled with water (23 ± 1℃) to a depth of 20 cm. Immediately after the swim, mice were removed from the tank and towel-dried before being placed back in home cages	2, 9, 17, 28
Food and water deprivation	Mice were subjected to 24 h of food and water deprivation. Food and water were provided immediately after the end of the fasting period	3, 8, 21, 27
Wet cage	200 ml water in 100 g bedding for 24 h. Immediately after the swim, mice were removed from the tank and towel-dried before being placed back in home cages	4, 11, 15, 24
Cage tilting	Cage tilting (45°) along the vertical axis for 24 h	5, 14, 20, 25
Restraint	Mice were individually restraint for 4 h inside 50 ml centrifuge tubes with proper holes for ventilation	6, 12, 16, 22
Shaking	Mice were shaking for 15 min in 150r/min	7, 13, 18, 26

### Behavioral evaluations

The test detail of open field test (OFT), sucrose preference test (SPT), forced swimming test (FST) and tail suspension test (TST) were added in supplementary file.

### Sample collection and measurement

The brain and hippocampus of d14 mice were collected at 0 h (wt-wt control, ko-wt control), 6 h (wt-wt 6 h, ko-wt 6 h) and 12 h (wt-wt 12 h, ko-wt 12 h) following LPS injection. Following a 4-week period of CUMS, mice were sacrificed via cervical dislocation. Serum was acquired by centrifuging the entire blood at speed of 5000 rpm for a duration of 20 min. Hippocampus were gathered and preserved at a temperature of − 80 °C for RNA extract, collected half brain samples were fixed using a 4% paraformaldehyde solution and embedded in paraffin.

### Histological and morphometric analysis

To access the activity and apoptosis of neurons in hippocampus, the paraffin tissue blocks that had undergone processing were sliced into 3 µm thick sections using a Leica microtome (Wetzlar, Germany) (n = 4–6 mice/group, with one section prepared per mouse. From each section, at least 2 fields of view were imaged and analyzed). Paraffin sections underwent automated deparaffinization and stained using the Haematoxylin and eosin (H&E) staining kit (Solarbio, Beijing, China), Nissl staining kit (Solarbio, Beijing, China) and TUNEL staining kit (LABLEAD, Beijing, China), separately. The positive-cell number and Nissl stain intensity were calculated using ImageJ software (NIH, Bethesda, MD, USA) (Zhu et al. 2021).

### Immunofluorescence

Paraffin sections were treated with a program to remove the paraffin, and then subjected to antigen retrieval using an enhanced citrate antigen retrieval solution (Beyotime Biotechnology, Jiangsu, China, Cat. No: P0083) under high temperature and pressure conditions. The sections were washed with PBS and permeabilized with 0.5% Triton X-100 at room temperature for 15 min. Next, the samples were blocked with an immune-blocking solution at room temperature for 1 h. Afterwards, the sections were left to incubate overnight at a temperature of 4 °C with primary antibodies (rabbit anti-IBA1, ABclonal, Wuhan, China, Cat. No: A19776, 1:100). After washing 3 times with PBS, followed by incubation for 1 h at room temperature with secondary antibodies (488-conjugated Goat Anti-Rabbit IgG, ABclonal, Wuhan, China, Cat. No: AS073, 1:1000) in a dark place. DAPI staining (Sigma, USA, Cat. No: D9542) was used to visualize the nuclei.

### Three-dimensional (3D) reconstruction

The deparaffinize sections were treated with anti-IBA1 for a duration of 24 h, and then stained with secondary antibody (Alexa Fluor 488). Imaging was conducted using a confocal microscope (Leica, Germany) with a 40× oil objective. Imaging parameters such as laser power, gain, and offset were kept consistent throughout the experiments. Stacking in the z direction was conducted with interval of 1.0 mm. The total process length was measured using the Neuron J plug-in in ImageJ software.

### Cell culture

The HT22 cells and BV2 cells were purchased from the *Institute of Basic Medical Sciences*, *Chinese Academy of Medical Sciences* (Beijing, China) and used between the 4th and 20th passages. These cells were maintained in Dulbecco's modified Eagle’s medium (DMEM) (Corning, NY) supplied with 10% FBS (VISTECH, New Zealand) and 1% penicillin–streptomycin mixture (Gibco) at 37 °C under a 5% CO_2_–95% air atmosphere in 25 cm^2^ plastic tissue culture flasks (Corning, NY).

### Cell proliferation and viability assays

HT22 cells were seeded in 96-well cell culture plates at a density of 5 × 10^3^ cells/well and cultured for 24 h in the complete culture medium. Then, the cells were exposed to increasing doses (0.1, 1, 10, 100, 1000 μg/mL) of RHLF (donated by Dr. Yunping Dai research group, China Agricultural University, China) for 2 days in complete culture medium, 10 μg/mL BSA as a control group. HT22 cells were exposed to increasing doses (0.5, 1, 2, 4 μg/mL) of LPS (Sigma, L6529, Escherichia coli, serotype O55:B5) for 24 h in complete culture medium, the complete culture medium without LPS as control.

HT22 cells were seeded in 96-well cell culture plates at a density of 5 × 10^3^ cells/well and cultured for 24 h in the complete culture medium. The cells were exposed to increasing doses (0.1, 1, 10, 100, 1000 μg/mL) of RHLF for 24 h, subsequently, 4 μg/mL LPS was added for 24 h.

Cell proliferation assays and cell activity assays were performed using the CCK8 assay kit (Beyotime Biotechnology, Jiangsu, China). Cell proliferation was calculated using the following formula: proliferation rate or cell activity (%) = average (absorbance treated with LF − blank)/average (absorbance treated without LF − blank) × 100%.

### BV2 cells migration assay

BV2 cells were seeded in 12-well cell culture plates at a density of 2 × 10^5^ cells/well and cultured for 24 h in the complete culture medium. Then, the cells were exposed to increasing doses (0.1, 10, 1000 μg/mL) of RHLF for 24 h in complete culture medium, 10 μg/mL BSA as a control group. A wound was created by scratching the cells using a sterile plastic pipette tip. At 0 h, pictures were taken. Then, the cells were exposed to LPS (1 μg/mL) or DMEM for 24 h. Subsequently, images were captured. the Image J software was used to determine cell migration based on the percentage of the wound closure area in three independent experiments.

### Phagocytosis assay

BV2 cells were plated on 24-well cell culture plates with cell climbing slice at a density of 5 × 10^4^ cells/well and cultured for 24 h in complete culture medium. The cells were incubated by increasing doses (0.1, 10, 1000 μg/mL) of RHLF for 24 h, 10 μg/mL BSA as a control group. Subsequently, 1 μg/mL LPS was added. After 24 h, pHrodo Red Zymosan Bioparticles (P35364, Invitrogen, Carlsbad, CA, USA) were added at a final concentration of 5 ng/µL (DMEM-dissolving) in 150 µL, incubated for 1 h in a humidified incubator with 5% CO2 at 37 °C, and then washed with DPBS for several times to remove excess beads. Use 4% PFA fixed cells, after washing, add closed incubation for 30 min, PBS was washed three times, and IBA1 antibody was added overnight at 4 °C. Finally, it was incubated with secondary antibody (Alex-488) at room temperature for 1 h. After PBS cleaning, the cell climbing slices were taken out and the number of beads per cell was measured for statistics using fluorescence microscope (Echo, USA).

### Quantitative real-time PCR

Total RNA of cells and hippocampus were extracted using an RNA extraction kit (Magen, Guangzhou, China). NanoDrop spectrophotometer (Thermo Fisher, Waltham, MA) was used to detect the quality of the RNA. By using the HiFiScript cDNA synthesis kit, reverse transcribed 1 μg of RNA into cDNA (NUOWEIZAN, Nanjing, China). Rotor-Gene Q (Qiagen, Hilden, Germany) with the Taq Pro Universal SYBR qPCR Master Mix Kit (NUOWEIZAN, Nanjing, China) was employed for RT-PCR. The RT-PCR amplification parameters consisted of initial denaturation at a temperature of 95 °C for a duration of 10 s, followed by annealing at a temperature of 55 °C for 30 s, and finally extension at a temperature of 72 °C for 32 s. We used glyceraldehyde-3-phosphate dehydrogenase (*Gapdh*) as the reference gene for normalization. Synbio Technologies (Jiangsu, China) synthesized the primer pairs, and their sequences can be found in Table 2.

**Table 2:** Sequences of primers for real-time PCR

Gene name	F-primer (5ʹ-3ʹ)	R-primer (5ʹ-3ʹ)
*Gapdh*	TCTCCTGCGACTTCAACA	TGTAGCCGTATT CATTGTCA
*TNF-α*	ACTGAACTTCGGGGTGATCG	CCACTTGGTGGTTTGTGAGTG
*IL-1β*	CTTCAGGCAGGCAGTATC	CAGCAGGTTATCATCATCATC
*IL-6*	ACAAAGCCAGAGTCCTTCAGAG	AGGAGAGCATTGGAAATTGGG
*IL10*	GGTGAGAAGCTGAAGACCCTC	CATGGCCTTGTAGACACCTTGG
*TLR4*	CACTGTTCTTCTCCTGCCTGA	GGAATGTCATCAGGGACTTTGC
*Myd88*	CGCATGGTGGTGGTTGTTTC	AGTCGCTTCTGTTGGACACC
*NFκB*	CCCTACGGAACTGGGCAAAT	GCAAATTTTGACCTGTGGGT
*IκBα*	CCTGACCTGGTTTCGCTCTT	CTGTATCCGGGTACTTGGGC
*Cd68*	CTGATCTTGCTAGGACCGCT	TGTGGCTGTAGGTGTCATCG
*Cd206*	CTGGAGTGATGGTTCTCCCG	GACATGCCAGGGTCACCTTT
*Bdnf*	TAAACGTCCACGGACAAGGC	AGTGTCAGCCAGTGATGTCG
*Grb2*	AACATCCGTGTCCAGGAACC	AAGTCTCCTCTGCGAAAGCC
*Camk1*	TGATCCTGGCAGAGGACAAGA	GCTACAATGTTGGGGTGCTTG
*Creb*	AACCAGCAGAGTGGAGATGC	GATGTTGCATGAGCTGCTGG

### Western blot

Proteins were extracted using RIPA lysis buffer (Beyotime Biotechnology, Jiangsu, China, Cat. No: P0013B) supplemented with protease inhibitor PMSF (Beyotime Biotechnology, Jiangsu, China, Cat. No:ST507) and phosphatase inhibitor (Roche, Basel, Switzerland, Cat.No:4906845001). Mixed protein samples and loading buffer at a ratio of 4:1 and boiled them for 10 min. After separating the proteins using SDS-PAGE (8–10%), the protein strip was transferred to a nitrocellulose membrane. To block the membranes, we used 5% non-fat milk powder in Tris-buffered saline and Tween (TBST) and incubated them with primary antibodies overnight at 4 °C. The following antibodies: Rabbit anti-p-ERK1/2 (Cell Signaling Technology, Cat. No: 4370), Rabbit anti-ERK1/2 (Cell Signaling Technology, Cat. No: 4695), Rabbit anti-Brain-derived neurotrophic factor (BDNF) (Abclonal, Cat. No: A11028), mouse anti-CREB1 (Santa Cruz Biotechnoloy, Cat. No: sc-377154), mouse anti-p-CREB1 (Santa Cruz Biotechnoloy, Cat. No: sc-81486), Rabbit anti-β-Tubulin (ABclonal, Cat. No: A12289), Rabbit anti-GAPDH (Abclonal, Cat. No: AC001). After washing three times with TBST, followed by incubation with secondary antibody for 1 h, Goat anti-rabbit IgG (ABclonal, Cat. No:AS014) or Goat anti-mouse IgG (ABclonal, Cat. No:AS003). Protein bands were detected using ECL (Beyotime, Cat. No:P0018S), and densitometry was performed by ImageJ software. The band intensity of each target protein was normalized to that of GAPDH/β-Tubulin.

### Statistic analyze

Mean ± SEM represents the expressed results. The statistical analysis was conducted utilizing IBM SPSS Statistic 23. Student’s t-test was used to compare two groups (*p < 0.05, **p < 0.01, and ***p < 0.001) and a one-way ANOVA, two-way ANOVA, three-way ANOVA and post hoc Duncan test were used to compare more than two groups, different letters stand for statistically significant each other (*p* < 0.05).

## Results

### LF-feeding deficiency 14-day-old mice exhibited a more drastic inflammatory response to LPS stimulation in early life

**Fig. 1:**
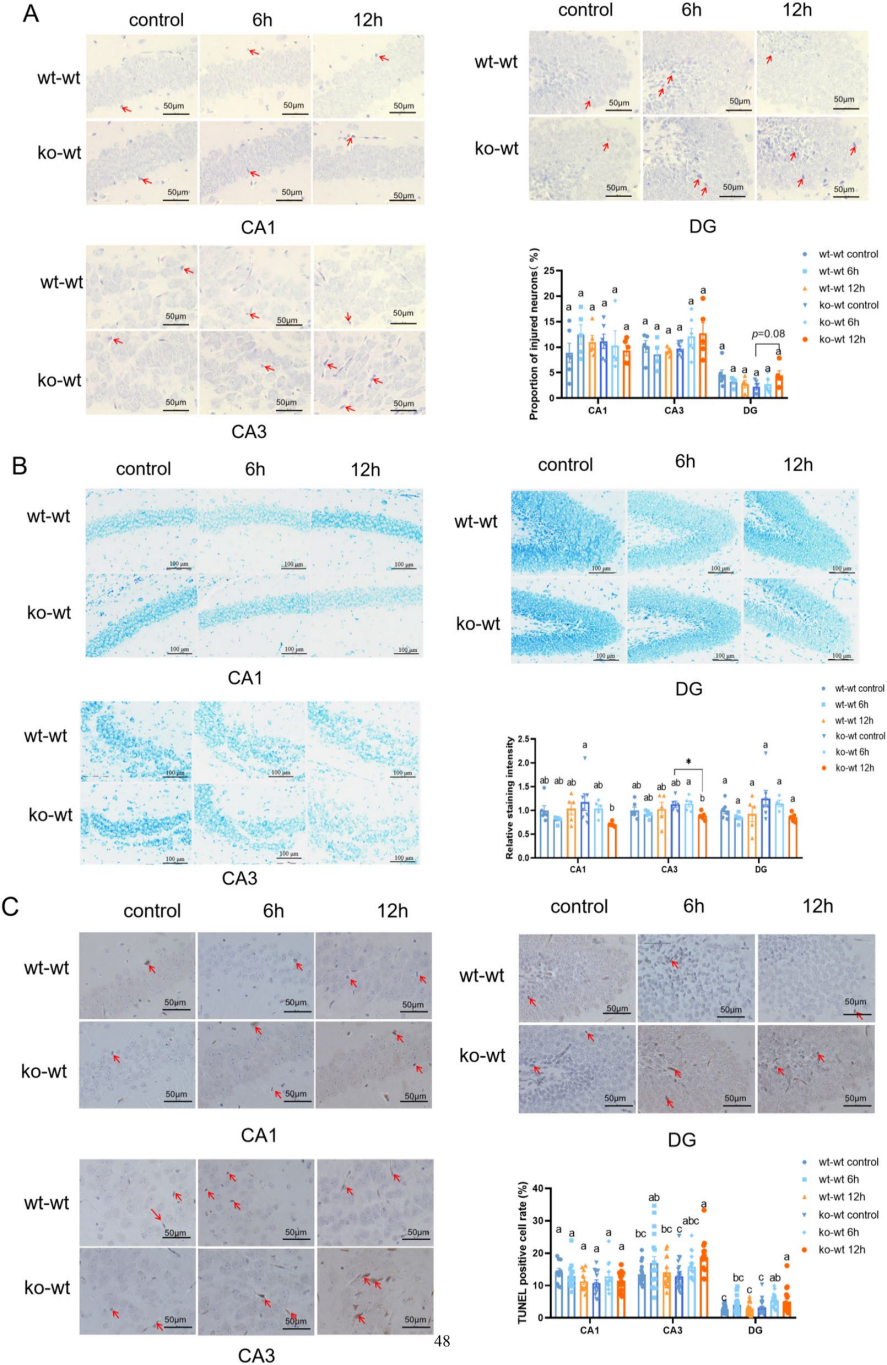
LF feeding deficiency during lactation increase hippocampal neuron damage in 14-day-old mice after injection of LPS. **A** HE staining of hippocampal CA1, CA3 and DG regions in 14-day-old mice after 6 and 12 h of LPS injection (magnification ×400). Proportion of injured neurons. Scale bars, 50 μm. Red arrowheads mark injured neurons, n = 4–6 (each point stands for one separate slice). **B** Nissl staining of hippocampal CA1, CA3 and DG regions in 14-day-old mice after 6 and 12 h of LPS injection (magnification × 200). Scale bars, 100 μm. Relative staining intensity (relative to wt-wt control group). n = 4–7 (each point stands for one separate slice). **C** TUNEL staining of hippocampal CA1, CA3 and DG regions in 14-day-old mice after 6 and 12 h of LPS injection (magnification ×400). Red arrowheads mark TUNEL-positive cells. The proportion of TUNEL positive cells was calculated. n = 9–27 (each point stands for one separate vision field). All data are presented as mean ± SEM. Student’s t-test was used to compare two groups (*p < 0.05, **p < 0.01, and ***p < 0.001) and a two-way ANOVA and post hoc Duncan test were used to compare more than two groups, different letters stand for statistically significant each other (*p* < 0.05)

According to Cao et al. (2021), day 14 is a critical time window during which inflammatory events can exert long-lasting effects on CNS development. The hippocampus plays a crucial role in emotion regulation, learning, and memory, and its dysfunction has been implicated in the development of depression and anxiety disorders. The hippocampus comprises the hippocampal gyrus, which encompasses the CA1, CA3, dentate gyrus (DG), and other regions. In this study, we examined the impact of LPS injections on hippocampal neuronal cell injury 6 and 12 h post-injection. Histological analysis using hematoxylin and eosin staining (Fig. 1A) revealed that there was no significant increase in the proportion of injured neurons (CA1, CA3, and DG) in either the wild-type (wt)-wt or knockout (ko)-wt groups. However, in the ko-wt group, the proportion of injured neurons in the hippocampal DG region showed an increasing trend (*p* = 0.08) 12 h after LPS injection. Histopathological changes in the neuronal cytoplasm and injuries were assessed using Nissl staining. Figure 1B demonstrates that the depth of Nissl staining in the ko-wt 12 h group was significantly reduced compared with that in the ko-wt control group in the hippocampal CA1 region. Additionally, the ko-wt 12 h group exhibited a noteworthy decrease in the depth of Nissl staining when compared with both the ko-wt control and ko-wt 6 h groups in the hippocampal CA3 region. No significant differences were observed in the DG. To further investigate LPS-induced apoptosis in the hippocampus, terminal deoxynucleotidyl transferase dUTP nick end labeling (TUNEL) staining was performed. TUNEL staining revealed no significant distinction observed among the six groups in the CA1 region (Fig. 1C). The number of apoptotic cells in the CA3 region was significantly higher in the ko-wt 12 h group compared with the ko-wt control and wt-wt 12 h groups (Fig. 1C). In the DG region, the TUNEL-positive cell rate in the ko-wt 6 h group was significantly higher than that in the ko-wt control mice, and the positive cell rate in the ko-wt 12 h group was higher than that in the ko-wt control group and the wt-wt 12 h group (Fig. 1C). In summary, at 14 days of age, mice deficient in LF during lactation exhibited significantly reduced neuronal activity in the hippocampal CA1 and CA3 regions along with an increased number of apoptotic cells in the CA3 and DG areas after 12 h of acute inflammatory stimulation. In contrast, mice with a normal LF intake during lactation did not show any significant changes in these indicators after inflammatory stimulation. These findings showed that the neuronal damage caused by LPS in the ko-wt groups was more obvious than that in the wt-wt groups.

**Fig. 2:**
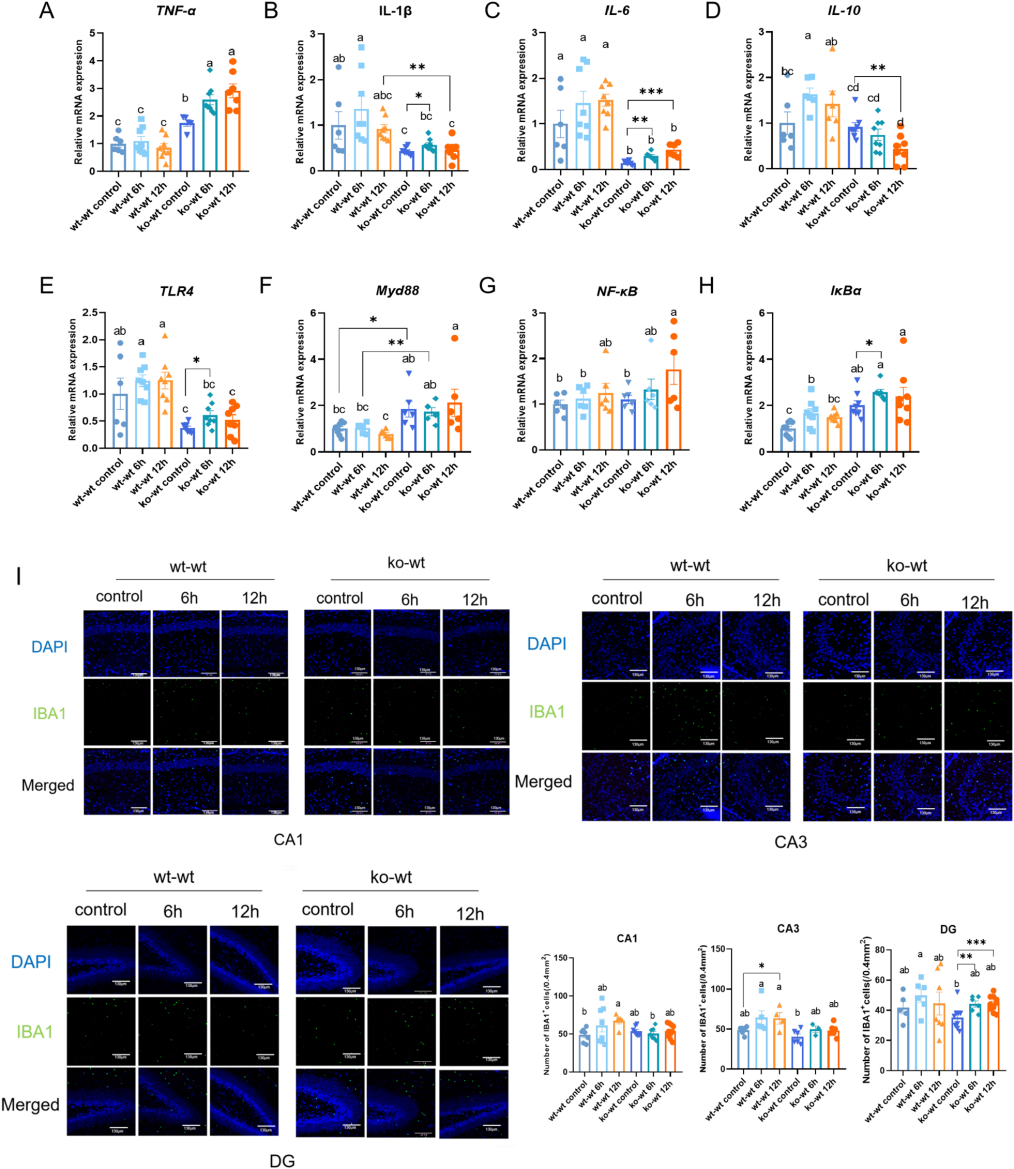
The hippocampal inflammation and microglial activation in 14-day-old mice after injection of LPS. **A**–**D** Relative mRNA expression of *TNF-α*, *IL-1β*, *IL6*, *IL10* in the hippocampus of 14-day-old mice after 6 and 12 h of LPS injection (relative to wt-wt control group). n = 6–8 (each point stands for one mouse). **E**–**H** Relative mRNA expression of *TLR4*-*NFκB* signal pathway in the hippocampus of 14-day-old mice after 6 and 12 h of LPS injection (relative to wt-wt control group). n = 6–8 (each point stands for one mouse). **I** Immunofluorescence analysis of IBA‐1 (green, a microglia marker) in hippocampus CA1, CA3 and DG regions of 14-day-old mice after 6 and 12 h of LPS injection (magnification ×100). Scale bars, 130 μm. n = 5–6 (each point stands for one separate slice). All data are presented as mean ± SEM. Student’s t-test was used to compare two groups (*p < 0.05, **p < 0.01, and ***p < 0.001) and a two-way ANOVA and post hoc Duncan test were used to compare more than two groups, different letters stand for statistically significant each other (*p* < 0.05)

Furthermore, an inflammatory response has been observed in the hippocampus. Figure 2A shows that the mRNA expression of tumor necrosis factor-alpha (TNF-α) in ko-wt mice (ko-wt control, ko-wt 6 h, and ko-wt 12 h groups) was significantly higher than in wt-wt mice (wt-wt control, wt-wt 6 h, and wt-wt 12 h groups). The mRNA expression of TNF-α increases with increasing elapsed time after LPS injection in ko-wt mice, but no notable change was found in wt-wt mice. Figure 2B and C shows that the mRNA expression of interleukin (IL)-1β and IL-6 in ko-wt mice was significantly lower than wt-wt mice (wt-wt control vs. ko-wt control, wt-wt 6 h vs. ko-wt 6 h, wt-wt 12 h vs. ko-wt 12 h). Six hours after LPS injection, the expression of IL-1β and IL-6 in ko-wt mice was significantly increased compared with the ko-wt control group. There was also an increasing trend in wt-wt mice, but the difference was not statistically significant. Twelve hours after LPS injection, the mRNA expression of IL-6 in ko-wt mice continued to increase, significantly surpassing that in the ko-wt control group. The wt-wt mice exhibited a similar pattern, albeit without statistical significance. Following LPS injection, the anti-inflammatory cytokine IL-10 showed a significant increase in the wt-wt 6 h group, whereas, in ko-wt mice, the expression of IL-10 declined progressively over time, reaching a significant decrease compared with the ko-wt control group after 12 h (Fig. 2D).

Activation of the Toll-like receptor 4 (TLR4)-myeloid differentiation primary response 88 (MyD88)-nuclear factor-kappa B (NF-κB) signaling pathway is associated with the release of LPS-induced inflammatory cytokines. The mRNA expression of TLR4 in ko-wt mice was significantly lower than that in wt-wt mice across all groups (wt-wt control vs. ko-wt control, wt-wt 6 h vs. ko-wt 6 h, wt-wt 12 h vs. ko-wt 12 h). TLR4 expression in the ko-wt 6 h group was significantly higher than that in the ko-wt control group (Fig. 2E). However, the mRNA expression of MyD88 in ko-wt mice was significantly higher than that in wt-wt mice across all groups (wt-wt control vs. ko-wt control, wt-wt 6 h vs. ko-wt 6 h, wt-wt 12 h vs. ko-wt 12 h), with no significant increase observed after LPS stimulation in either wt-wt or ko-wt mice (Fig. 2F). Under LPS stimulation, the expression of *NF-κB* exhibited a trend toward upregulation, with the ko-wt 12 h group significantly higher than the ko-wt control group (Fig. 2G). Six hours after LPS injection, the mRNA expression of IκBα was significantly increased in both wt-wt and ko-wt mice compared with their respective controls, with ko-wt mice still displaying a higher expression than wt-wt mice (Fig. 2H). These results suggest that LPS triggers a greater activation of the TLR4-MyD88-NF-κB signaling pathway, resulting in an increase in the expression levels of inflammatory factors in suckling mice with lactoferrin feeding deficiency.

Moreover, we evaluated the number of microglial cells as a possible measure of microglial activation in different regions of the hippocampus (Fig. 2I). In the CA1 region, there was a significant increase in the number of ionized calcium-binding adapter molecule 1 (IBA1)^+^ cells in the wt-wt 12 h group compared with that in the wt-wt control group, whereas no difference was observed in the ko-wt group. In the CA3 region, the number of IBA1^+^ cells in the wt-wt 12 h group was significantly higher than that in the wt-wt control group, and LPS increased microglial activation in ko-wt mice, although the difference was not statistically significant. In the DG region, LPS significantly increased microglial activation in ko-wt mice. In summary, early-inflammatory stimulation for 12 h increased the activation of microglia in the CA1 and CA3 regions of the hippocampus in suckling mice with normal LF intake and in the DG region of the hippocampus in suckling mice with LF deficiency.

### LF-feeding deficiency during lactation results in more serious depression in adults after early inflammation

**Fig. 3:**
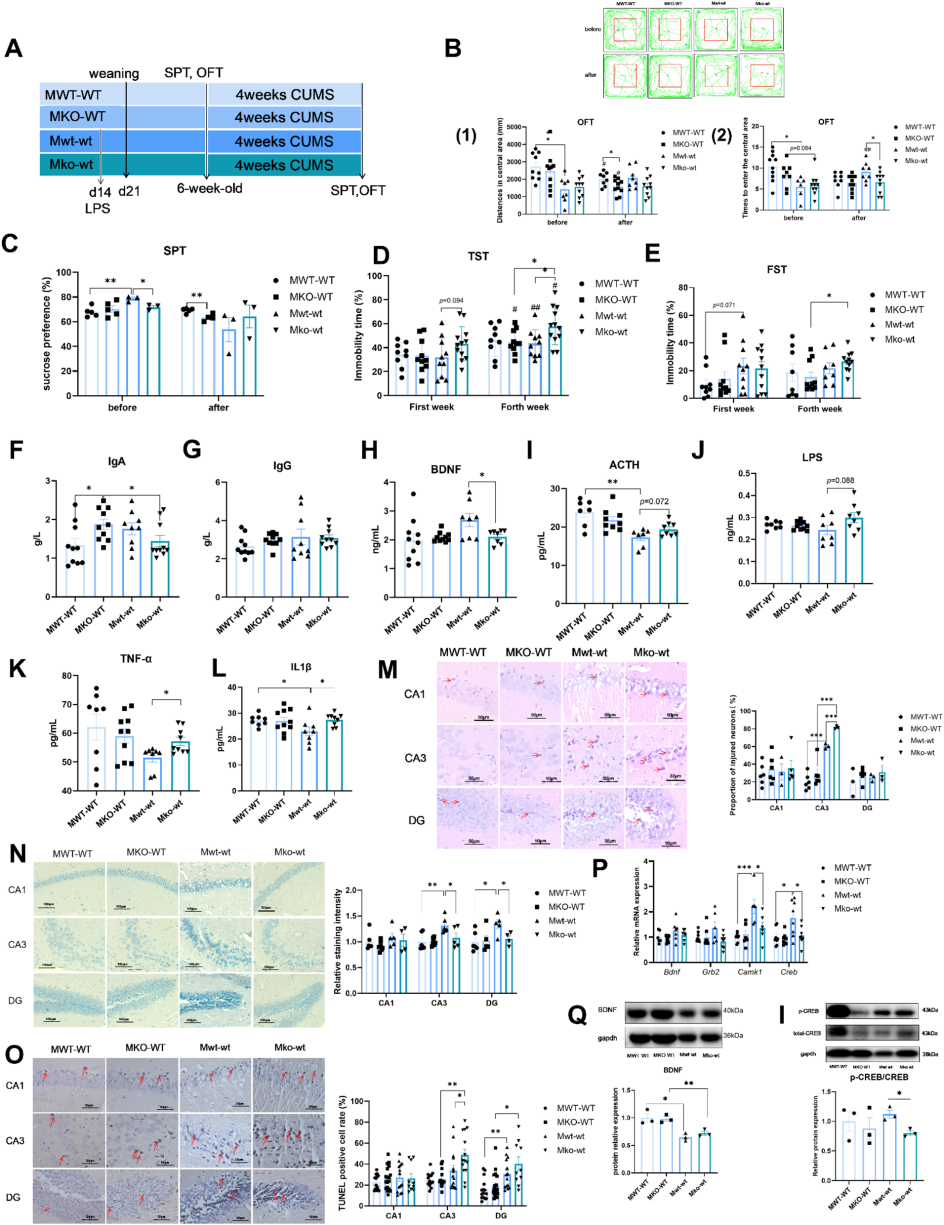
LF feeding deficiency during lactation increased the depressive behavior and neuronal damage in adult mice that suffered from early inflammatory challenge. **A** Schematic of the experimental protocol. **B** Motion trail, distances in central area and times to enter the central area of four group adult mice before and after CUMS in OFT. Green lines represent the motion trail, red point represent the start position of mice, blue point represent the end position of mice. n = 8–10 (each point stands for one mouse). **C** Sucrose preference of four group adult mice before and after CUMS. **D** Immobility time of four group adult mice before and after CUMS in TST. n = 8–10 (each point stands for one mouse). **E** Immobility time of four group adult mice before and after CUMS in FST. n = 8–10 (each point stands for one mouse) (**B**–**E**), All data were tested for between-group effects with three-way ANOVA analysis. Student’s t-test was used to compare two groups (*p < 0.05, **p < 0.01, and ***p < 0.001), # represent a significant difference compared with before CUMS (#*p* < 0.05, ##*p* < 0.01). **F** Content of IgA, **G** IgG, **H** BDNF, **I** ACTH, **J** LPS, **K** TNFα, **L** IL-1 β in serum of four group adult mice after CUMS. **F**–**L** n = 8–10 (each point stands for one mouse). **M** HE staining in hippocampus of four group adult mice after CUMS (magnification ×400). Scale bars, 50 μm. Proportion of injured neurons, n = 3–6 (each point stands for one mouse). Red arrowheads mark injured neurons. **N** Nissl staining in hippocampus of four group adult mice after CUMS (magnification ×200). Scale bars, 100 μm. Relative staining intensity (relative to MWT-WT group), n = 4–7 (each point stands for one separate slice). **O** TUNEL staining in hippocampus of four group adult mice after CUMS (magnification ×400). Red arrowheads mark TUNEL-positive cells. Scale bars, 50 μm, n = 10–19 (each point stands for one separate vision field). **P** Relative mRNA expression of* BDNF*-*CREB* signaling pathway in hippocampus of four group adult mice after CUMS. n = 6–8 (each point stands for one mouse). **Q** Western blot analysis of BDNF expression in hippocampus of four group adult mice after CUMS, n = 3 (each point stands for one mouse). **I** Western blot analysis of p-CREB/CREB expression in hippocampus of four group adult mice after CUMS, n = 3 (each point stands for one mouse). **F**–**I** Two- way ANOVA analysis was performed between four group (MWT-WT, MKO-WT, Mwt-wt, Mko-wt). Student’s t-test was used to compare two groups (*p < 0.05, **p < 0.01, and ***p < 0.001). All data are presented as mean ± SEM

To further explore the effect of early inflammation on adult depression in mice with LF-feeding deficiency, we conducted a study using 6-week-old male mice. Both groups, one without early-inflammatory stimulation (MWT-WT, MKO-WT) and the other with early-inflammatory stimulation (Mwt-wt, Mko-wt), were subjected to CUMS to induce depression for 4 weeks (Fig. 3A).

The results of the three-way analysis of variance showed that the effect of early-inflammatory stimulation on the distance in the open-field central zone was significant (F (1,65) = 11.155, *p* = 0.001 < 0.01). Additionally, an interaction between early-inflammatory stimulation and CUMS modeling was observed (F (1,65) = 12.939, *p* = 0.001 < 0.01). Before CUMS, the distance in the center square of the open field was significantly reduced in mice with early-inflammatory stimulation compared with those without (Mwt-wt vs. MWT-WT, *p* = 0.003 < 0.01). After CUMS, the distance in the central area of MWT-WT (*p* = 0.025 < 0.05) and MKO-WT (*p* = 0.045 < 0.05) mice was significantly decreased compared with the distance before CUMS, and the distance in the central area of MKO-WT mice was significantly shorter than that in the MWT-WT group (*p* = 0.048 < 0.05) (Fig. 3B(1)). However, in mice stimulated by early inflammation (Mwt-wt and Mko-wt), there was no significant change in the distance of the central area after CUMS compared with that before CUMS, which may be associated with the thresholds of the open-field test and CUMS. The distance in the central area of the Mko-wt group was lower than that of the Mwt-wt group; however, the difference was not significant (*p* = 0.111) (Fig. 3B(1)). The number of times they entered the central area also reflected the degree of anxiety in mice (Zhu et al. 2021). Similar to Fig. 3B(1), the results of the three-way ANOVA showed that the effect of early-inflammatory stimulation on the times entered the central area was significant (F (1,62) = 4.113, *p* = 0.047 < 0.05), and there was an interaction between early-inflammatory stimulation and CUMS (F (1,62) = 14.336, *p* = 0.000 < 0.001). Before CUMS, early-inflammatory stimulation decreased the time to enter the center area (Mwt-wt vs. MWT-WT, *p* = 0.01 < 0.05; Mko-wt vs. MKO-WT, *p* = 0.084 > 0.05), but no difference was found between LF-feeding mice and LF deficiency mice (Fig. 3B(2)). After CUMS, the time to enter the center area in the Mwt-wt group was significantly longer than that before CUMS (*p *= 0.002 < 0.01), and the time to enter the center area in the Mko-wt group was significantly lower than that in the Mwt-wt group (*p* = 0.042 < 0.05) (Fig. 3B(2)). The results of the three-way ANOVA showed that CUMS significantly influenced sucrose preference in mice (F (1,23) = 11.033, *p* = 0.003 < 0.01), and there was an interaction between early-inflammatory stimulation and CUMS (F (1,23) = 5.535, *p* = 0.028 < 0.05). Additionally, the interaction between early-inflammatory stimulation, LF feeding, and CUMS was significant (F (1,23) = 5.031, *p* = 0.035 < 0.05). Mice without LF intake during the suckling period and stimulated by inflammation (Mko-wt) showed a lower sucrose preference than Mwt-wt mice before CUMS (*p* = 0.021 < 0.05). However, the sucrose preference of Mwt-wt mice was significantly higher than that of MWT-WT mice (*p* = 0.007 < 0.01) (Fig. 3C). After CUMS, the sucrose preference of MKO-WT mice was significantly lower than that of MWT-WT mice (*p* = 0.008 < 0.01), indicating that LF-feeding deficiency mice exhibited more severe depressive behaviors (Fig. 3C). In the tail suspension test (Fig. 3D), the impact of LF intake during lactation (F (1,74) = 4.023, *p* = 0.049 < 0.05) and CUMS modeling (F (1, 74) = 17.308,* p* = 0.000 < 0.001) on immobility time was significant. Additionally, there was an interaction between lactoferrin intake and early-inflammatory stimulation (F (1,74) = 5.452, *p *= 0.022 < 0.05). The immobility time of the Mko-wt group was longer than that of the Mwt-wt group (*p* = 0.094 > 0.05) before CUMS. After CUMS, the immobility time of Mko-wt mice was significantly longer than that of Mwt-wt (*p* = 0.024 < 0.05) and MKO-WT mice (*p* = 0.032 < 0.05), and the immobility time of MKO-WT (*p* = 0.019 < 0.05), Mwt-wt (*p *= 0.008 < 0.01), and Mko-wt (*p* = 0.01 < 0.05) mice was significantly longer than that before CUMS. However, no difference was found between the MWT-WT and MKO-WT mice, either before or after CUMS, in the absence of early-inflammatory stimulation. In the forced swim test (Fig. 3E), mice stimulated with early inflammation showed a longer immobility time before CUMS; however, the difference was not significant (Mwt-wt vs. MWT-WT, *p* = 0.071 > 0.05; Mko-wt vs. MKO-WT, *p* = 0.468 > 0.05). After 4 weeks of CUMS, the immobility time of Mko-wt mice was significantly longer than that of MKO-WT mice (*p *= 0.013 < 0.05); however, this difference was not observed in LF-feeding mice.

After CUMS, serum indicators of the four groups of mice were determined. Serum IgA levels (Fig. 3F) in the MKO-WT group were significantly higher than those in the MWT-WT and Mko-wt groups (*p* = 0.024 < 0.05,* p* = 0.041 < 0.05). The IgG levels did not differ among the four groups (Fig. 3G). Brain growth factor is widely recognized, and its level in Mko-wt mice was lower than that in Mwt-wt mice (*p* = 0.039 < 0.05) (Fig. 3H). The level of adrenocorticotropic hormone and LPS (Fig. 3I, J) also showed an increased trend in the Mko-wt group compared with the Mwt-wt group (*p* = 0.072 > 0.05, *p* = 0.088 > 0.05), and the interaction between early-inflammation stimulation and lactoferrin feeding influences the levels of adrenocorticotropic hormone (F (1,27) = 4.714, *p* = 0.039 < 0.05) and LPS concentration (F (1,30) = 3.978, *p* = 0.055 > 0.05). The amounts of inflammatory cytokines, such as TNF-α (Fig. 3K) and IL-1β (Fig. 3L), in the Mko-wt group were significantly higher than in the Mwt-wt group (*p* = 0.015 < 0.05, *p* = 0.028 < 0.05). The level of IL-1β in the Mwt-wt group was significantly lower than in the MWT-WT group (*p* = 0.042 < 0.05). These results suggest that early-inflammatory stimulation causes depressive symptoms in mice before the establishment of a depression model and that mice exposed to early inflammation and LF deficiency during lactation displayed more severe depression after CUMS.

In the present study, we assessed the hippocampal neuronal damage. The results of hematoxylin and eosin staining showed that the number of damaged neurons in the CA3 area of depressed mice stimulated by early inflammation was significantly higher than that in mice without early-inflammatory stimulation (Mwt-wt vs. MWT-WT, *p* = 0.000 < 0.001; Mko-wt vs. MKO-WT, *p* = 0.000 < 0.001), and the proportion of damaged neurons in the Mko-wt group was significantly higher than that in the Mwt-wt group (*p *= 0.000 < 0.001) (Fig. 3M). However, no significant differences were observed between CA1 and DG regions. Nissl staining suggested that the Mko-wt group had more severe neuronal injury than the Mwt-wt group in the CA3 and DG regions (*p* = 0.049 < 0.05, *p* = 0.037 < 0.05, respectively). However, the Nissl staining intensity of the Mwt-wt group was significantly higher than that of the MWT-WT in the CA3 and DG regions (*p* = 0.006 < 0.01, *p* = 0.016 < 0.05, respectively) (Fig. 3N). Hippocampal apoptosis was also assessed. TUNEL staining indicated no variation in the number of apoptotic cells in the CA1 region among the four groups (Fig. 3O). In the CA3 region, the Mko-wt group exhibited a significantly greater number of apoptotic cells than both the Mwt-wt (*p *= 0.05) and MKO-WT (*p* = 0.001 < 0.01) mice. The number of cells undergoing apoptosis in the DG of mice with early inflammation was considerably greater than that in non-inflammatory mice (Mwt-wt vs. MWT-WT, *p* = 0.003 < 0.01; Mko-wt vs. MKO-WT,* p* = 0.013 < 0.05). The results of our study suggest that adult mice lacking the lactating LF experience increased hippocampal neuronal damage when exposed to early-inflammatory stimulation, which is linked to depressive symptoms.

Brain-derived neurotrophic factor (BDNF) is a growth factor that plays essential roles in neuronal development and synaptic plasticity. The BDNF signaling pathways activate cAMP-response element binding protein (CREB), which controls genes associated with neural plasticity (Zhong et al. 2019). The levels of calcium/calmodulin-dependent protein kinase type 1 (*Camk1*) and growth factor receptor-bound protein (*Creb*) mRNA expression in the Mko-wt group were significantly lower than those in the Mwt-wt group (*p* = 0.042 < 0.05, *p* = 0.029 < 0.05, respectively), and the mRNA expression levels of *Bdnf* and *Creb* 2 in the Mko-wt mice were not significantly different from those in the Mwt-wt group (Fig. 3P). At the protein level, BDNF expression in mice stimulated with early inflammation was significantly lower (Mwt-wt vs. MWT-WT, *p *= 0.000 < 0.001; Mko-wt vs. MKO-WT, *p* = 0.016 < 0.05). Two-factor ANOVA revealed a significant effect of early inflammation (F (1,8) = 6981.643,* p* = 0.008 < 0.01) and LF intake during lactation (F (1, 8) = 205.474,* p* = 0.044 < 0.05) on BDNF levels (Fig. 3Q). CREB phosphorylation was significantly lower in Mko-wt mice than in Mwt-wt mice (*p* = 0.04 < 0.05) (Fig. 3I).

### The mice with LF-feeding deficiency and early-inflammation exposure exhibited significant microglial activation and inflammation in the hippocampus during adult depression

**Fig. 4:**
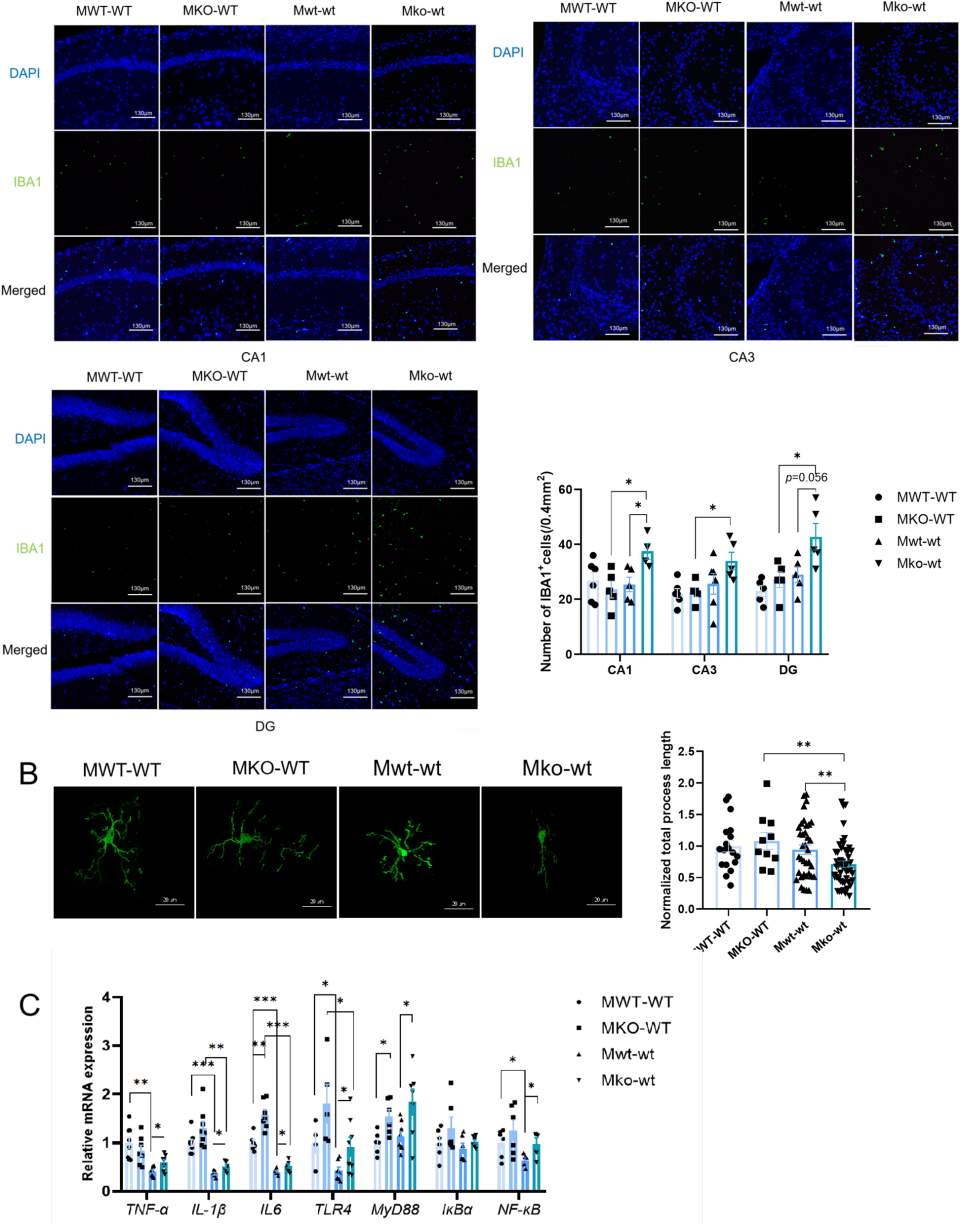
LF feeding deficiency during lactation increased microglial activation and inflammation in the hippocampus of adult mice that suffered from early inflammatory challenge. **A** Immunofluorescence analysis of IBA‐1 (green, a microglia marker) in hippocampus CA1, CA3 and DG regions of four group adult mice after CUMS (magnification ×100). Scale bars, 130 μm, n = 5–6 (each point stands for one mouse). **B** Representative images 3D reconstruction of hippocampus microglia in four group adult mice after CUMS. Scale bars, 20 μm. Normalized total process length, n = 10–50 (each point stands for one microglia). **C** Relative mRNA expression of *TNF-α*, *IL-1β*, *IL6* and *TLR4*-*NFκB* signal pathway in hippocampus of four group adult mice after CUMS, n = 4–8 (each point stands for one mouse). Two-way ANOVA analysis was performed for between-group effects. Two-tailed Student’s t-test was used to compare two groups (*p < 0.05, **p < 0.01, and ***p < 0.001). All data are presented as mean ± SEM

Neuronal damage triggers microglial activation, leading to an inflammatory response (Ignatenko et al. 2018). Over-activated microglia release molecules that induce neuronal damage, thereby increasing depression levels (Zhang et al. 2020). Immunofluorescence assays revealed that the number of activated microglia in the CA1 region was notably higher in the Mko-wt group than in the Mwt-wt and MKO-WT groups (*p* = 0.034 < 0.05, *p* = 0.016 < 0.05), and a significant interaction effect was observed between early-inflammation stimulation and LF feeding (F (1,13) = 6.274,* p* = 0.026 < 0.05) (Fig. 4A). In the CA3 area of the mouse hippocampus, the impact of LF feeding on the number of IBA1^+^ cell was significant (F (1,12) = 6.222, *p* = 0.028 < 0.05), the number of IBA1^+^ cell in the Mko-wt group was notably higher than in MKO-WT mice (*p *= 0.036 < 0.05) and a significant interactive effect was observed between early-inflammation stimulation and LF feeding in the CA3 region (F (1,12) = 5.583,* p *= 0.036 < 0.05) (Fig. 4A). In the DG region of the hippocampus, both early-inflammatory stimulation and LF feeding significantly influenced the number of IBA1 + cells (F (1,12) = 8.194,* p* = 0.014 < 0.05; F (1,12) = 9.835, *p* = 0.009 < 0.01). The number of IBA1^+^ cells was significantly higher in the Mko-wt group than in MKO-WT (*p* = 0.026 < 0.05) and Mwt-wt (*p* = 0.056 > 0.05) mice. Semi-automatic quantitative morphometric 3D measurements of microglia showed that Mko-wt mice had significantly shorter processes than the MKO-WT and Mwt-wt groups (*p *= 0.005 < 0.01, *p* = 0.099 < 0.01), indicating a higher microglial activation status in Mko-wt mice (Fig. 4B).

Furthermore, mRNA levels of inflammatory cytokines and the TLR4-NFκB signaling pathway in the hippocampus were detected. The mRNA expression of *TNF-α* and *NF-κB* in the Mwt-wt group was significantly lower than in the MWT-WT group (*p* = 0.001 < 0.01, p = 0.03 < 0.05). The Mko-wt group had significantly higher mRNA expression levels of *TNF-α* and *NF-κB* than the Mwt-wt group (*p* = 0.027 < 0.05, *p* = 0.043 < 0.05). The mRNA expression of *IL-1β*, *IL-6*, and *TLR4* in mice stimulated by early inflammation was significantly lower than that in non-inflammatory mice, and the expression of *IL-1β* and *TLR4* in the Mko-wt mice was significantly higher than in the Mwt-wt mice (*p* = 0.035 < 0.05,* p* = 0.048 < 0.05). LF-feeding-deficient mice had significantly higher levels of *IL-6* and *Myd88* than LF-feeding mice (MKO-WT vs. MWT-WT, Mko-wt vs. Mwt-wt). Taken together, these results suggest that under inflammatory stimulation, microglial activation and inflammation in various regions of the hippocampus are more severe in lactoferrin-feeding-deficient mice after adult CUMS modeling.

### RHLF attenuates LPS-induced neuronal damage and microglial activation in vitro

**Fig. 5:**
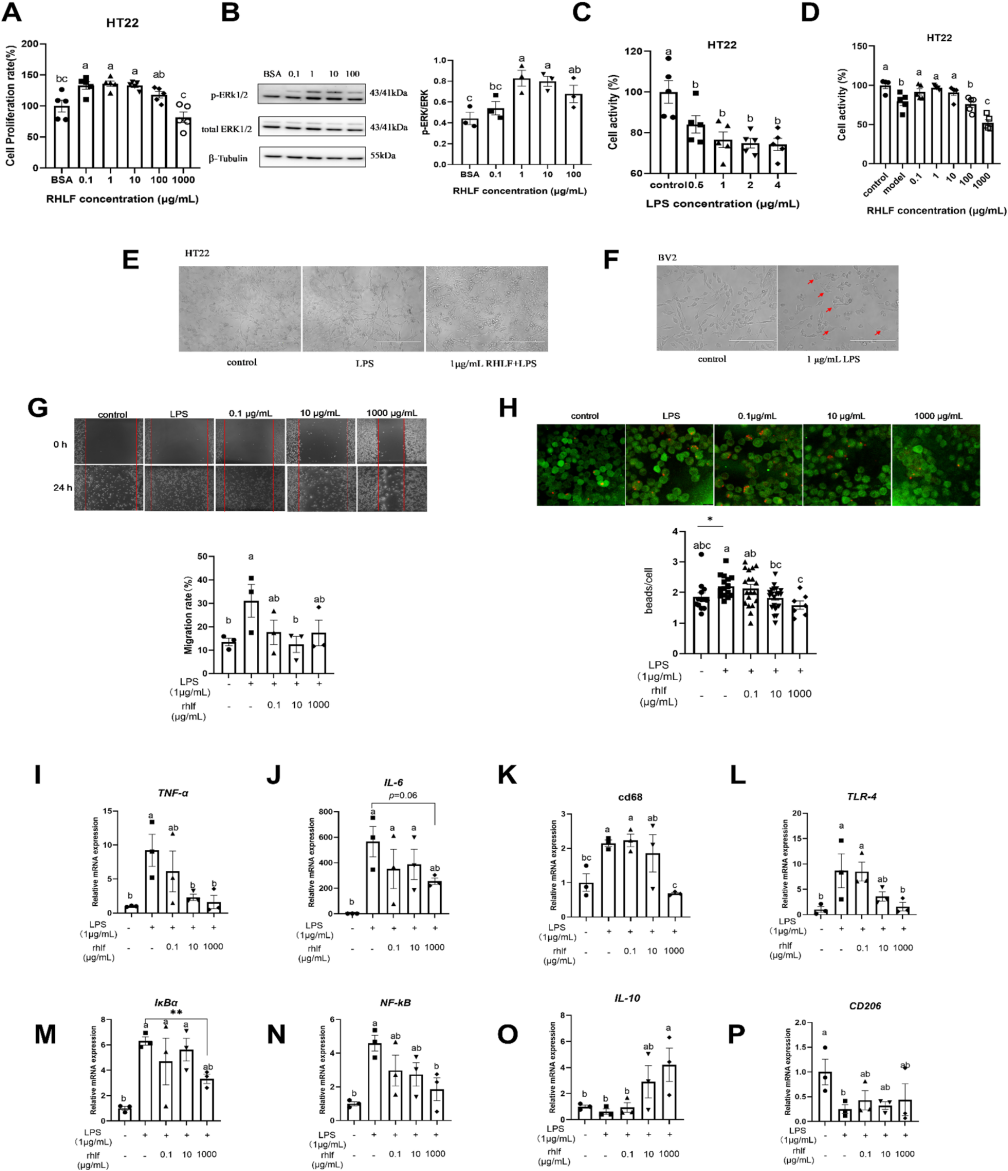
RHLF promotes HT22 proliferation and prevents LPS-induced HT22 neuronal damage and BV2 microglia activation. **A** Effects of RHLF on the proliferation of HT22 cells treated for 48 h, n = 5 (each point stands for one separate cell culture wells). **B** Western blot analysis of p-ERK/ERK in HT22 cells treated by RHLF, n = 3 (each point stands for one separate cell culture wells). **C** Effects of LPS on HT22 cell activity, n = 5 (each point stands for one separate cell culture wells). **D** Protective effect of RHLF on the activity of HT22 cells damaged by LPS, n = 3–5 (each point stands for one separate cell culture wells). **E** The morphology of HT22 was observed using phase morphology (magnification ×100). Scale bars, 400 μm. **F** The impact of LPS on the morphology of BV2 cells (magnification ×200). Scale bars, 200 μm. **G** Effect of RHLF on migration of BV2 cells induced by LPS, n = 3 (each point stands for one separate cell culture wells), (magnification ×40). **H** Effect of RHLF on phagocytosis of BV2 cells induced by LPS, n = 7–23 (each point stands for one separate BV2 cell), (magnification ×200, Scale bars, 20 μm). **I**–**K** Effect of RHLF on the mRNA expression of BV2 cells M1 marker, n = 3 (each point stands for one separate cell culture wells). **L**–**N** Effect of RHLF on the mRNA expression of *TLR4*-*NFκB* signal pathway, n = 3 (each point stands for one separate cell culture wells). **O**, **P** Effect of RHLF on the mRNA expression of BV2 cells M2 marker, n = 3 (each point stands for one separate cell culture wells). One-way ANOVA and post hoc Duncan test was performed for the multi group analysis, different letters stand for statistically significant each other (*p* < 0.05). Two-tailed Student’s t-test was performed for the two-group analysis (*p < 0.05, **p < 0.01, and ***p < 0.001). All data are presented as mean ± SEM. All the n value in this figure represents an independent biological sample

To further investigate the protective function of LF against LPS-induced neuronal damage and microglial activation, we used a concentration gradient of RHLF to treat an in vitro-cultured mouse hippocampal neuronal HT22 cell line and mouse microglia BV2 cell line. The results showed that 0.1–10 µg/mL RHLF significantly promoted the proliferation of HT22 cells after 2 days of treatment (Fig. 5A). Furthermore, 1–100 µg/mL RHLF promoted extracellular signal-regulated protein kinases 1 and 2 (ERK1/2) phosphorylation, a known regulator of cell proliferation (Wang et al. 2022), as shown in Fig. 5B. The cell activity and conditions were notably reduced following treatment with 0.5–4 µg/mL LPS. To establish the LPS-damage model, we selected the treatment of 4 µg/mL for 1 day (Fig. 5C). Significantly, the decline in cell activity induced by LPS was effectively alleviated by RHLF at concentrations ranging from 0.1–10 µg/mL, while a higher concentration of RHLF (1000 µg/mL) was observed to exacerbate the impairment of cell activity caused by LPS (Fig. 5D), the representative microscopy results are shown in Fig. 5E, indicating that RHLF significantly protects HT22 cells from LPS-induced cell death.

Consistent with reported results (Li et al. 2016), upon treatment with 1 µg/mL LPS for 24 h, the BV2 spherical cell bodies became larger, and the cell processes contracted, indicating a morphological transformation into an activated state (Fig. 5F). The LPS concentration was determined by referencing previously published articles (Zhang et al. 2017; Harun et al. 2015; Li et al. 2016; Gaire et al. 2013). Microglial cell movement and phagocytic activity are associated with pro-inflammatory responses (Nam et al. 2018). Consequently, we investigated whether RHLF influenced the LPS-induced migration and phagocytic activity of BV2 microglial cells. These findings suggested that the migration of BV2 microglia was significantly enhanced by LPS treatment compared with the control treatment (Fig. 5G). Furthermore, the presence of 10 µg/mL RHLF resulted in a significant reduction in the migration of BV2 microglial cells stimulated by LPS, as observed in Fig. 5G. To assess the phagocytic capability of the BV2 cells, the number of fluorescent beads engulfed by the cells was measured. In the LPS group, the number of beads engulfed by each cell type was considerably higher than in the control group. However, when exposed to 10 and 1000 µg/mL RHLF, the number of beads phagocytosed was significantly reduced compared with the LPS-stimulated group (Fig. 5H).

The mRNA expression of M1 markers in BV2 cells was measured, as illustrated in Fig. 5I–K. LPS significantly increased the expression of *TNF-α* in BV2 cells, and pretreatment with 10 µg/mL and 1000 µg/mL of RHLF significantly reduced the expression of *TNF-α* (Fig. 5I). LPS also significantly increased the mRNA expression of *IL-6* and *CD68* in BV2 cells, and pretreatment with 1000 µg/mL of RHLF reduced the expression of *IL-6* (*p* = 0.06) and *CD68* (Fig. 5J, K). Subsequently, the activation of the TLR4-NF-κB signaling pathway was determined. LPS significantly increased the expression of *TLR4*, *IκBα*, and *NF-κB* in BV2 cells, while pretreatment with 1000 µg/mL of RHLF significantly inhibited the LPS-induced increase in *TLR4*, *IκBα*, and *NF-κB* expression (Fig. 5L, M, N). Finally, the expression of M2 markers in the BV2 cells was measured. Compared with the control group, LPS stimulation had no effect on the expression of *IL-10*, but 1000 µg/mL of RHLF significantly increased the expression of *IL-10* compared with the LPS-treated group (Fig. 5O). LPS stimulation significantly decreased the expression of *CD206* compared with the control group, but RHLF did not have any impact on the expression of *CD206* (Fig. 5P).

## Discussion

The inadequate integration of lactoferrin into formulas has resulted in breastfeeding being the primary method for infants to receive lactoferrin supplementation on a broader scale (Zhao 2016). In low- and middle-income nations, the exclusive breastfeeding rate for children under 6 months of age is only 37%, which is even lower in high-income countries (Victora et al. 2016). Consequently, a significant number of infants may experience lactoferrin deficiency during breastfeeding; however, no epidemiological studies have assessed or quantified this phenomenon. Therefore, it is crucial to explore the effects of lactoferrin deficiency during lactation on the long-term well-being of individuals, with a specific focus on its potential impact on depression.

From a clinical perspective, lactoferrin exhibits noticeable protective effects against neonatal inflammatory illnesses. Several randomized controlled trials have shown that lactoferrin supplementation in preterm infants with extremely low birth weight can lead to a significant decrease in the prevalence of fungal infections, sepsis, and neonatal necrotizing enterocolitis (Alfaleh 2012; Manzoni et al. 2014). Preclinical indications suggest that lactoferrin has neuroprotective effects in the developing brain by alleviating neuronal injury, improving cerebral connectivity, stimulating the production of neurotrophic factors, and diminishing inflammation in models of perinatal inflammatory stimulation, intrauterine growth restriction, and neonatal hypoxic-ischemia (Schirmbeck et al. 2022). Our study revealed that LF-deficient mice exhibited increased neuronal damage and significant inflammatory responses to LPS stimulation in the hippocampus. In the ko-wt group, Nissl bodies, which play a crucial role in neuronal protein synthesis and functional status, showed a gradual decrease in staining depth as the LPS stimulation time increased, indicating a continuous decline in neuronal metabolic capacity (Fig. 1B). TUNEL staining was performed to further assess the occurrence of LPS-induced apoptosis. The number of apoptotic cells increased with LPS stimulation in LF mice, whereas no such increase was observed in lactoferrin-fed mice (Fig. 1C).

*NF-κB* is recognized for its role in regulating the transcriptional activity of pro-inflammatory cytokine genes (*IL-1β*, *IL-12*, *TNF-α*, *IL-6* and *IL-8*) (Lingappan et al. 2013). LF has the ability to be internalized within the cell and transported to the nucleus, where it hinders cytokine production by interfering with the intracellular processes that lead to *NF-kB* activation (Håversen et al. 2002). Our findings demonstrate that the expression levels of *TNF-α*, *IL-1β*, and *IL-6* remained unchanged following LPS injection in wt-wt mice (Fig. 2A–C). In ko-wt mice, the basal levels of *IL-1β*, *IL-6*, and *TLR4* were significantly lower compared to wt-wt mice (ko-wt control vs. wt-wt control). This observation may be attributed to the role of lactoferrin in facilitating the natural development of the immune system. The neonatal immune system undergoes a crucial maturation during lactation. Lactoferrin enhances the activation of innate immune cells, and the absence of lactoferrin during lactation leads to delayed maturation of the neonatal mouse immune system (Legrand 2012). Typically, the reduced activity of natural immune cells is commonly characterized by diminished expression of cytokines (IL-1β and IL6), chemokines, and pattern recognition receptor (TLR4) (Li 2014). The increase of TNF-α, MyD88, and IκBα (ko-wt control vs. wt-wt control) may serve as a compensatory mechanism to uphold the body's immune function. A study found that LF enhances mRNA expression levels of different interleukins in a physiological context, including IL-1β, IL-8, and IL-10 (Prenner et al. 2007). Consistent with our study, LF enhanced the expression of IL-1β and reduced the production of TNF-α in the spleen of piglets, although the difference did not reach statistical significance (Li 2014). Longitudinal expression of the anti-inflammatory factor IL-10 demonstrated the impact of lactoferrin on immune homeostasis in the nervous system. In wt-wt mice, the initiation of the anti-inflammatory phase was detected at 6 h post-LPS stimulation, implying a swift resolution of the inflammatory stimulus and gradual recovery of CNS homeostasis. In contrast, in mice with LF feeding deletion, the levels of anti-inflammatory factors remained low even after 12 h of LPS stimulation, indicating a prolonged and intensified pro-inflammatory state. Prolonged inflammation can exacerbate neuronal damage (Tian et al. 2012).

The release of inflammatory factors into the nervous system is closely associated with microglial activation (Tian et al. 2012). In our study, we observed that LPS-activated microglia to varying extents in specific subregions of 14-day-old hippocampi (Fig. 2I). Indeed, experiments in mice have revealed that the transcriptome of microglia and their susceptibility and response to injuries vary depending on the region (Kreisel et al. 2018). In our study, microglia in the CA1 and CA3 regions of wt-wt mice were rapidly activated (Fig. 2I). During acute injury, the rapid activation of microglia plays a protective role on neurons by engulfing cellular waste and inhibiting inflammatory responses (Neumann et al. 2006; Lana et al. 2020). The protective function was also found in our results. The histopathological examination of the hippocampus showed that there was no significant damage in the CA1 and CA3 areas stimulated by LPS in the wt-wt group (Fig. 1A–C). In the mammalian hippocampal DG, granule neurons are continuously produced from neural stem cells throughout their life cycle and are integrated into the hippocampal network (Lattanzi et al. 2020). Microglia in the DG display a distinct transcriptome profile compared with other hippocampal regions, such as CA1, as revealed by whole-genome analyses (Kreisel et al. 2018). Lana et al. discovered that astrocytes and microglia in the DG of LPS-treated rats engage in the phagocytosis of apoptotic granule neurons. Moreover, the activation of DG microglia in ko-wt mice (Fig. 2I) could be associated with an elevated rate of apoptosis in granule neurons (Fig. 1C). Our results show that early inflammatory stimulation activated microglia in different hippocampal regions in both LF-feeding and LF-deficient mice. We speculate that this may be related to the interregional heterogeneity in microglia (Lana et al. 2020; Grabert et al. 2016). Differences in bioenergetic and immunoregulatory pathways were the major sources of heterogeneity (Grabert et al. 2016). Our previous research indicates that lactoferrin during lactation significantly affects the transcription of immune-related genes in the hippocampus of 18-day-old mice (Wang et al. 2023), this may contribute to the microglial heterogeneity observed across different hippocampal regions in our current study.

Combined with the performance of lactation LF-feeding-deficient mice in early-inflammatory stimulation, this study further examined the depressive behavior of lactation LF-feeding-deficient mice in adulthood. The results indicated that lactation LF-deficient mice displayed an increased depressive phenotype subsequent to inflammatory stimulation during early life and CUMS modeling in adulthood (Fig. 3B–E). Considering the role of LF in immune system development, we measured serum antibody levels (Fig. 3F, G). Under identical stimulation conditions, the IgA levels in the MKO-WT group were significantly higher than those in the MWT-WT group, indicating that mice lacking LF in their diet showed a more intense adaptive immune response, which may be related to the decline in natural immune function induced by lactoferrin deficiency (Wang et al. 2023). The CA1 region of the hippocampus plays a crucial role in cognitive functions, particularly learning and memory. Its role is vital in integrating temporal aspects and preserving short-term memory. In contrast, the CA3 region is responsible for the rapid formation of spatial and contextual memory. Numerous studies have indicated that the hippocampal CA3 neurons display increased responsiveness to stressors (Tata and Anderson 2010; Gourley et al. 2013). Depression is closely linked to diminished neurogenesis, which is primarily regulated by the DG region (Travis et al. 2015). In our study, the hippocampal CA3 and DG regions were severely damaged in Mko-wt mice (Fig. 3M–O). Neural plasticity is a fundamental mechanism of neuronal adaptation, which can be enhanced by increasing BDNF levels. The role of BDNF signaling in the pathophysiology of depression and the mechanisms underlying antidepressant treatment are of great importance (Zhang et al. 2016). Our findings indicated that early inflammation may be impairing neuroplasticity in mice (Fig. 3Q). Similar to previous studies (Chen et al. 2015), mice fed LF exhibited higher activation of the BDNF signaling pathway in adulthood (Fig. 3P, I). Previous studies of the mechanisms underlying depression have predominantly focused on neuronal dysfunction. However, the impact of microglia on depression has been extensively investigated in recent years. Our research revealed a remarkable surge in the activation of hippocampal microglia in lactoferrin-deficient mice (Mko-wt) compared with that in mice administered lactoferrin (Fig. 4A, B). Cao et al. found that inflammation during early life enhances microglial responsiveness to stressful stimuli (Cao et al. 2021), and this phenomenon was observed in LF-deficient mice but not in LF-fed mice (Fig. 4A, B, Mko-wt vs. MKO-WT). However, the ko-wt group exhibited reduced activation of hippocampal microglia compared with the wt-wt group in 14-day-old mice (Fig. 2I). The connection between excessive microglial activation in adulthood and early inflammation in lactoferrin-deficient mice prompts speculation about the potential neuroplastic damage resulting from early inflammation. The absence of lactoferrin exacerbates the impairment of neuronal plasticity in the hippocampus owing to early inflammation, thereby increasing the risk of neuronal damage upon exposure to adult CUMS, which can trigger microglial activation. However, further experiments are required to validate this hypothesis. Additionally, our previous research has shown that lactoferrin deficiency during lactation increases gut microbiota dysregulation in adult mice (Wang et al. 2023). The microbiota-gut-brain axis is known to be an important regulator of glial functions (Loh et al. 2024), and gut microbiota and metabolites can directly influence microglial inflammatory responses (Li et al. 2021; Zheng et al. 2023). Therefore, the observed effects of lactoferrin deficiency on microglia may also be related to gut microbiota alterations rather than a direct action of lactoferrin itself. We plan to investigate this possibility in greater depth in future studies. Our study revealed that the expression of pro-inflammatory factors in the serum and hippocampus of mice subjected to early-inflammatory stimulation was significantly lower than that in mice without such stimulation (Figs.3K, L and 4C). This phenomenon may be associated with immune tolerance. When individuals with immune tolerance were exposed to a second inflammatory stimulus, there was a decrease in the expression of pro-inflammatory cytokines and TLR4 (Alves-Rosa et al. 2002). Additionally, immune tolerance was more pronounced in LF-fed mice than in those that were not fed LF. However, further investigation is necessary to elucidate this phenomenon.

To validate the role of lactoferrin in neurons and microglia, in vitro experiments were performed. Numerous studies have demonstrated the substantial influence of LF on the regeneration of bones, muscles, and skin, primarily through the facilitation of the ERK signaling pathway (Wang et al. 2022). Our study showed that RHLF stimulated the proliferation of hippocampal precursor neurons (HT22 cells) by upregulating ERK1/2 phosphorylation (Fig. 5B). In addition, based on our in vivo results, RHLF protected neurons from LPS-induced damage. A certain level of microglial activation is necessary for tissue repair, but excessive activation can lead to neuronal cell death. To investigate the specific impact of LF on microglial overactivation under pathological conditions while minimizing confounding factors from the immune system, we subjected BV2 cells to LPS treatment to induce microglial activation and inflammation. The stimulation of microglia by LPS is associated with increased migration and phagocytosis, leading to chronic inflammation and neuronal damage. In response, RHLF significantly reduced LPS-induced microglial migration and phagocytosis (Fig. 5G, H). RHLF played a crucial role in suppressing the expression of M1 markers (Fig. 5I–K) in microglia by downregulating the TLR4 signaling pathway (Fig. 5L–N). Studies have indicated that TLR4 is crucial for activated microglia to cause neuronal damage in living organisms (Dai et al. 2015). TLR4-deficient mice were used to prepare mixed CNS cultures, and LPS did not induce neuronal injury (Dai et al. 2015). Furthermore, RHLF exhibits a tendency to promote the transformation of microglia into the M2 phenotype (Fig. 5O, P). Furthermore, recent reports indicate a correlation between the M2 microglial phenotype and reduced damage, as well as possible restoration (Orihuela et al. 2016; Manzoni et al. 2018).

## Conclusions

**Fig. 6:**
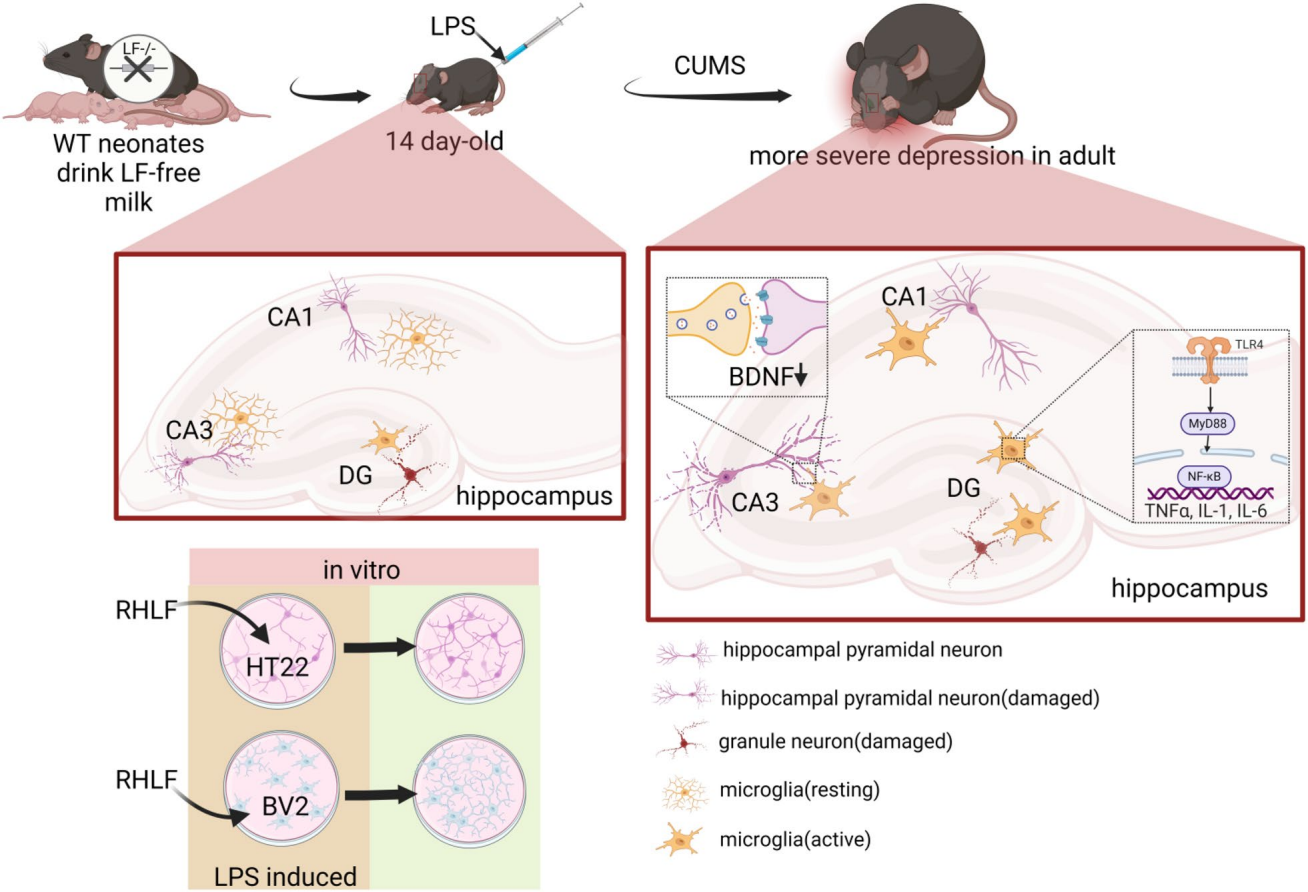
Graphical summary of the study

In conclusion, our study indicates that LF consumption during lactation may positively influence adult depression by modulating microglial activation, alleviating neuroinflammation, and preserving neuronal integrity. Conversely, the absence of lactoferrin consumption during lactation hinders the prompt activation of microglia in the hippocampus in response to early acute inflammation in mice, thereby exacerbating the progression of inflammation and neuronal damage. Moreover, mice with impaired lactoferrin intake display an increased susceptibility to depressive symptoms in adulthood following early-inflammatory stimulation, which can be attributed to neuronal damage and excessive activation. In vitro, RHLF can promote neuronal proliferation and reduce LPS-induced neuronal damage and microglial activation. A graphical summary of this study is shown in Fig. 6.

## Supplementary Information

Supplementary Material 1.

## Data Availability

No datasets were generated or analysed during the current study.

## Abbreviations

LF: Lactoferrin
LPS: Lipopolysaccharide
TLR4: Toll-like receptor 4
NF-κB: Nuclear transcription factor kappa B
RHLF: Recombinant human lactoferrin
TNF-α: Tumour necrosis factor-α
IL: Interleukin
CUMS: Chronic unpredictable mild stress
KO: Knockout
WT: Wild-type
OFT: Open field test
TST: Tail suspension test
SPT: Sucrose preference test
FST: Forced swimming test
H&E: Haematoxylin and eosin
3D: Three-dimensional
DMEM: Dulbecco’s modified Eagle’s medium
Gapdh: Glyceraldehyde-3-phosphate dehydrogenase
TBST: Tris-buffered saline and tween
DG: Dentate gyrus
BDNF: Brain-derived neurotrophic factor
NSCs: Neural stem cells
