# Supplementary material for: Lactoferrin alleviates the adverse effects of early-life inflammation on depression in adults by regulating the activation of microglia
Source: Mol Med. 2025 Feb 7;31:50. doi: 10.1186/s10020-025-01094-9 (PMC11803964; doi:10.1186/s10020-025-01094-9)
Supplement: Supplementary file 1 — Supplementary Material 1. [file 10020_2025_1094_MOESM1_ESM.docx]

**
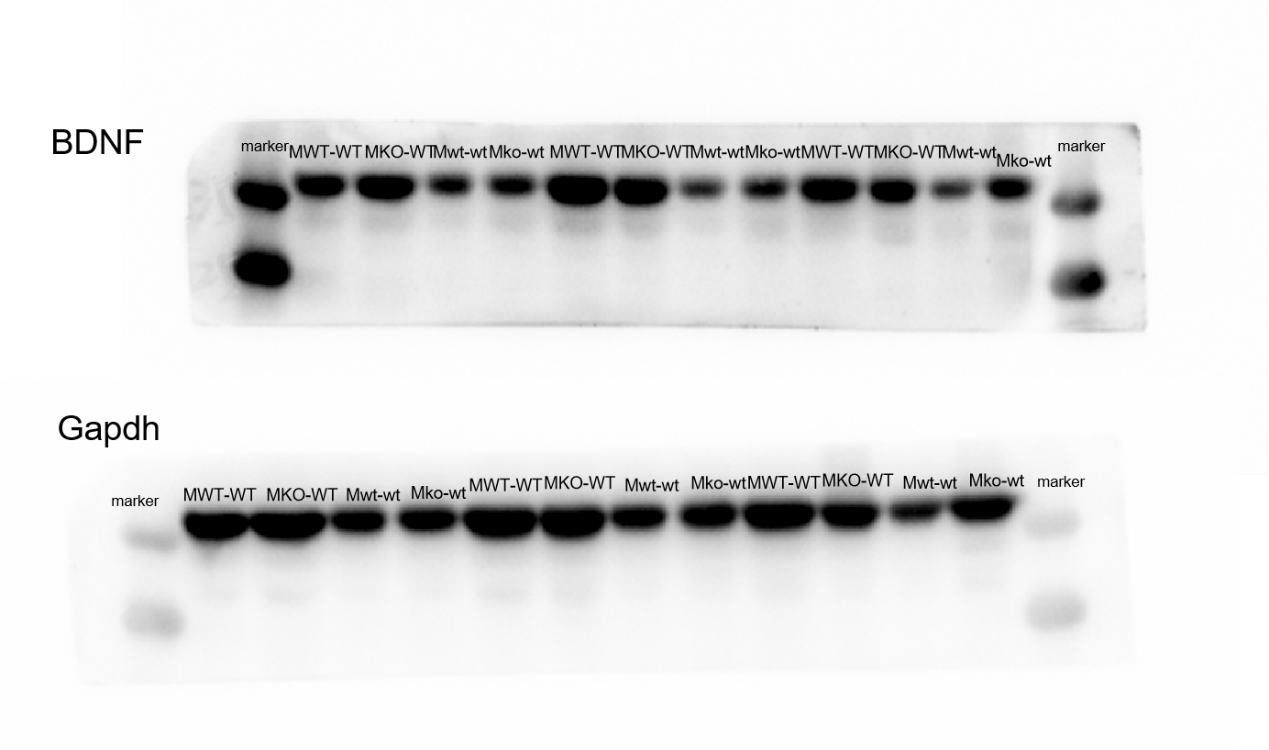
**

Fig. S1 The uncropped blot images of Fig. 3Q

**
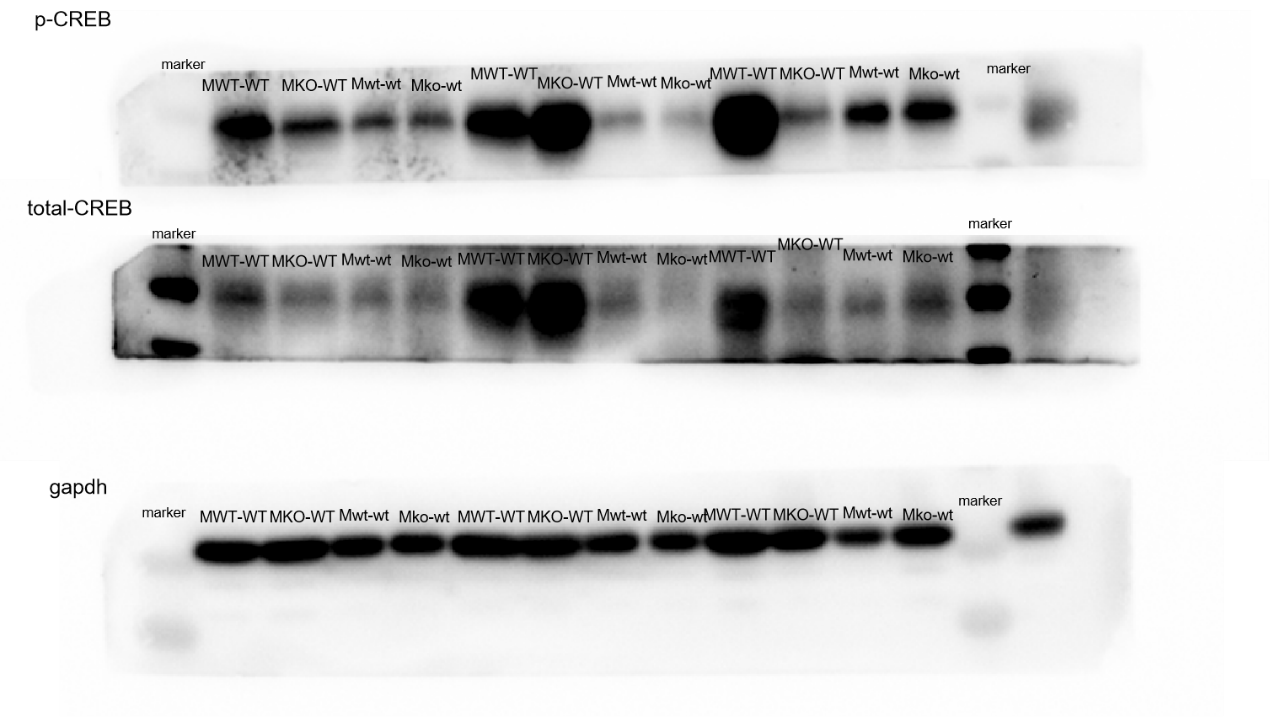
**Fig. S2 The uncropped blot images of Fig. 3I

**
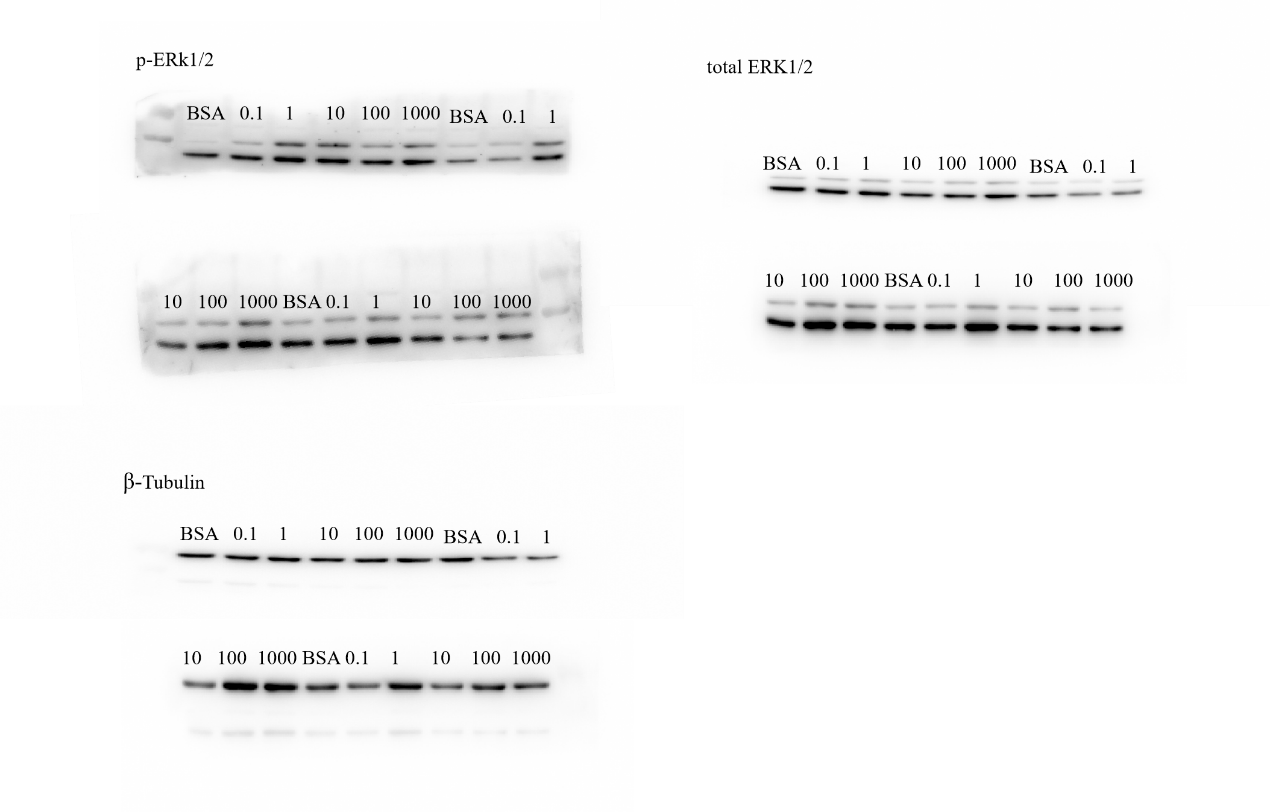
**

Fig. S3 The uncropped blot images of Fig. 5B

Behavioral evaluations

1.Open field test (OFT)

The test consisted of one 5 min trial in a white opaque 50 cm × 50 cm × 50 cm arena. The center zone was defined as a 25 cm × 25 cm central square. To begin each test, a mouse was introduced to the center of the square and its behavior was captured on video (Samsung, South Korea) over the course of 5min, the area was cleaned with 50% ethanol and allowed to dry completely between each test. The distance that each mouse walked in either the peripheral or central regions was quantified using KEmaze software.

2 .Sucrose preference test (SPT)

The sucrose preference test was performed according to a previous report [12] with minor modifications. Before the test, mice were acclimatized to sucrose solution. At first, two bottles of 1% sucrose solution (w/v) were placed in each cage for 24 h to avoid sucrose neophobia. After adaptation, mice were deprived of liquid and food for 24 h and then given the sucrose preference test. Each mouse had free access to 2 bottles for 24 h: one bottle with 1% sucrose solution (w/v) and another bottle with tap water. To avoid position effects, the positions of the 2 bottles were reversed at intervals of 12 h. Sucrose consumption and water consumption were measured by comparing the weights of the bottles before and after the test. The sucrose preference was calculated as sucrose preference (%) = (sucrose solution intake)/ (sucrose solution intake+ tap water intake) *100%.

3. Forced swimming test (FST)

Each mouse was placed in an open cylindrical container (diameter 10 cm, height 30 cm) with 23±1℃ water to a depth of 20 cm and allowed to swim for 6 min. Immobility times were measured during the last 4 min of the test. During the test, the behavior of each mouse was recorded using a video camera (Samsung, South Korea), and immobility time was measured using SuperFst software (KEWBIO Co., Ltd. Nanjing, China).

4. Tail suspension test (TST)

Mice were habituated to the testing room for 30 min before the experiments. Animals were attached by their tails to a shelf with medical adhesive tape (placed approximately 1 cm from the distal end of the tail). The last 4 min was recorded for immobility time during a 6-min test. During the test, the behavior of each mouse was recorded using a video camera (Samsung, South Korea), and immobility time was measured using SuperTst software (KEWBIO Co., Ltd. Nanjing, China).
